# A back-up mechanism for replication

**DOI:** 10.7554/eLife.107379

**Published:** 2025-05-29

**Authors:** Godefroid Charbon, Anders Løbner-Olesen

**Affiliations:** 1 Department of Biology, University of Copenhagen Copenhagen Denmark

**Keywords:** DNA replication, replication initiation, replication origin, helicase loading, primosome, replication stress, *E. coli*

## Abstract

A protein called PriC allows DNA replication to proceed in *Escherichia coli* when the complex that usually initiates this process is compromised.

**Related research article** Yoshida R, Korogi K, Wu Q, Ozaki S, Katayama T. 2025. Primosomal protein PriC rescues replication initiation stress by bypassing the DnaA-DnaB interaction step for DnaB helicase loading at *oriC*. *eLife* **13**:RP103340. doi: 10.7554/eLife.103340.

Ensuring that DNA replication occurs at the right stage of the cell cycle is of crucial importance in biology. In bacteria, DNA replication begins with the assembly of a DNA-protein complex called the orisome (Grimwade and Leonard, 2021). Formation of this complex is a multistep process that starts with molecules of the initiator protein DnaA binding to a region of the chromosome known as the origin of replication (*oriC*), and culminates in the formation of two DNA/DnaA subcomplexes.

Each subcomplex forms on an array of binding sites called DnaA boxes (Figure 1). One subcomplex opens the double helix at a region known as the DNA unwinding element, while the other helps extend and stabilize the unwound region, resulting in short stretches of single-stranded DNA (Kasho et al., 2023). Finally, each subcomplex helps to load enzymes called helicases onto each single strand, which then recruit the rest of the cellular components needed to synthesize DNA (Frimodt-Møller et al., 2024).

**Figure 1.**
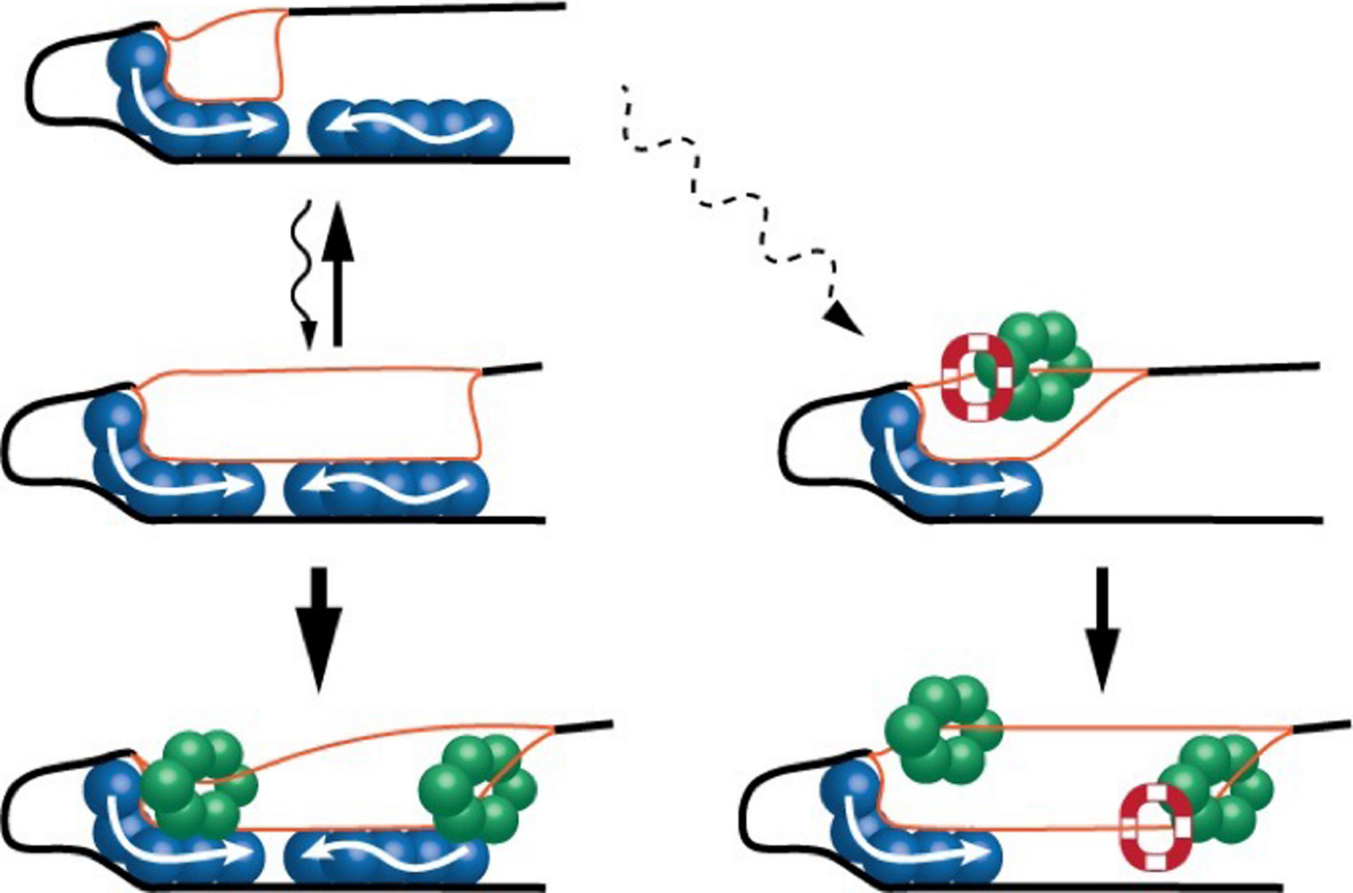
A simplified model of helicase loading in bacteria. (Top left) In bacteria, DNA replication starts with the formation of two DNA/DnaA subcomplexes (blue) at the origin of chromosome replication (*oriC*). One subcomplex (left) unwinds a section of the DNA double helix (black line) to form short segments of single-stranded DNA (orange lines). (Middle left) The second subcomplex helps extend the length of the unwound region and stabilize the single-stranded DNA. (Bottom left) Helicases (green) can be loaded onto the single-stranded DNA and replication can proceed. (Middle right and bottom) Occasionally, formation of the second subcomplex is disrupted: when this happens, a protein called PriC (red and white) can step in and help extend the length of the unwound region, allowing helicases to be loaded onto the single-stranded DNA.

Most of what is known about the orisome has come from studying the bacterium *Escherichia coli*. Surprisingly, these bacteria are viable without the regulatory circuits known to control the orisome, provided that the cells are not subjected to stress (Boesen et al., 2024). Similarly, *E. coli* can survive even when orisome assembly is disrupted, but their fitness is impaired and they are less robust to environmental stress.

These findings led researchers to propose that *E. coli* has an alternative, back-up mechanism for initiating replication (Hinds and Sandler, 2004). Previous studies have suggested that a protein called PriC may be involved, as it has been shown to restart stalled cycles of replication (Michel and Sandler, 2017). Now, in eLife, Shogo Ozaki, Tsutomu Katayama and co-workers from Kyushu University Graduate School of Pharmaceutical Sciences – including Ryusei Yoshida as first author – report how PriC takes over when the orisome fails to form (Yoshida et al., 2025).

Orisome assembly can be compromised by a wide range of factors, such as mutations in *oriC*, environmental changes, antimicrobial exposure, viral infections, and disruptions in the ratio of components in the complex. Some viruses that infect bacteria even produce proteins that block DNA replication by specifically interfering with the assembly of the orisome (Noguchi and Katayama, 2016).

To investigate how PriC compensates for these disruptions, Yoshida et al. blocked different stages of orisome assembly. This revealed that when DnaA was prevented from loading helicases onto *oriC*, replication could only continue if PriC was present. Additional experiments showed that PriC could also rescue replication when DnaA-helicase interactions were impaired, or when the parts of the *oriC* sequence needed for helicase loading were deleted. Taken together, these findings suggest that PriC takes over the task of helicase loading when orisome formation is compromised. The only requirement for this rescue mechanism to work is that DnaA must form the initial sections of single-stranded DNA.

A key insight from this study is that PriC only needs one of the two DnaA/DNA subcomplexes to be present to initiate replication, even though both are essential for full orisome assembly. This suggests that decades of mutagenesis studies may need to be re-evaluated, as experiments performed in *E. coli* strains that contain PriC might have underestimated the requirements for DnaA-dependent helicase loading.

This finding also raises a new question: if a single subcomplex is sufficient for initiation, why do most bacteria have two or more arrays of DnaA binding sites (Wegrzyn and Konieczny, 2023)? A possible answer to this question is that a single long array of DnaA binding sites would be too efficient at initiating DNA replication and would thus lead to uncontrolled replication and cell division. As such, it may be that *oriC* is deliberately sub-optimally organized as a way to control the cell cycle.

Based on the findings of Yoshida et al., one can imagine a model in which a single short DNA/DnaA subcomplex initiates the process of unwinding the DNA, but in the absence of the second subcomplex, it cannot stabilize the opened region (Figure 1). This prevents the subcomplex from unwinding enough of the DNA to load helicases onto the single strands. However, this partial unwinding is sufficient for PriC to take on this role, allowing *E. coli* to occasionally bypass the need for a fully assembled orisome.

The study by Yoshida et al. offers important insights into the role of PriC in DNA replication. While PriC is less efficient at initiating replication than the orisome, it allows *E. coli* to continue dividing and remain viable during unfavorable conditions. Further work could investigate whether similar back-up mechanisms exist in other bacterial species – or even in organisms beyond the bacterial kingdom.
